# Traumatic Brain Injury and Subsequent Risk of Brain Cancer in US Veterans of the Iraq and Afghanistan Wars

**DOI:** 10.1001/jamanetworkopen.2023.54588

**Published:** 2024-02-15

**Authors:** Ian J. Stewart, Jeffrey T. Howard, Eduard Poltavskiy, Michael Dore, Megan E. Amuan, Krista Ocier, Lauren E. Walker, Karl C. Alcover, Mary Jo Pugh

**Affiliations:** 1 Department of Medicine, Uniformed Services University of Health Sciences, Bethesda, Maryland; 2 Military Cardiovascular Outcomes Research Program, Uniformed Services University of Health Sciences, Bethesda, Maryland; 3 Miltary & Health Research Foundation, Laurel, Maryland; 4 Department of Public Health, University of Texas, San Antonio; 5 Department of Medicine, Duke University, Durham, North Carolina; 6 Informatics, Decision-Enhancement, and Analytic Sciences Center of Innovation, VA Salt Lake City Health Care System, Salt Lake City, Utah; 7 Division of Epidemiology, Department of Internal Medicine, University of Utah School of Medicine, Salt Lake City; 8 The Henry M. Jackson Foundation for the Advancement of Military Medicine Inc, Bethesda, Maryland

## Abstract

**Importance:**

While brain cancer is rare, it has a very poor prognosis and few established risk factors. To date, epidemiologic work examining the potential association of traumatic brain injury (TBI) with the subsequent risk of brain cancer is conflicting. Further data may be useful.

**Objective:**

To examine whether a history of TBI exposure is associated with the subsequent development of brain cancer.

**Design, Setting, and Participants:**

A retrospective cohort study was conducted from October 1, 2004, to September 20, 2019, and data analysis was performed between January 1 and June 26, 2023. The median follow-up for the cohort was 7.2 (IQR, 4.1-10.1) years. Veterans Affairs (VA) and Department of Defense (DoD) administrative data on 1 919 740 veterans from the Long-Term Impact of Military-Relevant Brain Injury Consortium–Chronic Effects of Neurotrauma Consortium were included.

**Exposure:**

The main exposure of interest was TBI severity (categorized as mild, moderate or severe [moderate/severe], and penetrating).

**Main Outcomes and Measures:**

The outcome of interest was the development of brain cancer based on *International Classification of Diseases, Ninth Revision, Clinical Modification (ICD-9-CM)* or *International Statistical Classification of Diseases, Tenth Revision, Clinical Modification (ICD-10-CM)* diagnostic codes in either the DoD/VA medical records or from the National Death Index.

**Results:**

After 611 107 exclusions (predominately for no encounter during the study period), a cohort including 1 919 740 veterans was included, most of whom were male (80.25%) and non-Hispanic White (63.11%). Median age at index date was 31 (IQR, 25-42) years. The cohort included 449 880 individuals with TBI (mild, 385 848; moderate/severe, 46 859; and penetrating, 17 173). Brain cancer occurred in 318 individuals without TBI (0.02%), 80 with mild TBI (0.02%), 17 with moderate/severe TBI (0.04%), and 10 or fewer with penetrating TBI (≤0.06%). After adjustment, moderate/severe TBI (adjusted hazard ratio [AHR], 1.90; 95% CI, 1.16-3.12) and penetrating TBI (AHR, 3.33; 95% CI, 1.71-6.49), but not mild TBI (AHR, 1.14; 95% CI, 0.88-1.47), were associated with the subsequent development of brain cancer.

**Conclusions and Relevance:**

In this cohort study of veterans of the Iraq and Afghanistan wars, moderate/severe TBI and penetrating TBI, but not mild TBI, were associated with the subsequent development of brain cancer.

## Key Points

**Question:**

Is traumatic brain injury (TBI) associated with the subsequent risk of brain cancer in Iraq and Afghanistan veterans?

**Findings:**

In this cohort study of more than 1.9 million veterans, moderate or severe and penetrating TBI were associated with the subsequent development of brain cancer. However, mild TBI was not associated with later brain cancer diagnoses.

**Meaning:**

The findings of this study suggest that moderate/severe and penetrating TBI are potentially novel risk factors for brain cancer in veterans.

## Teaser

This cohort study assesses whether traumatic brain injury is associated with subsequent diagnosis with brain cancer among Iraq and Afghanistan veterans.

## Introduction

Primary brain cancer is a relatively rare diagnosis, occurring in 7.02 per 100 000 persons in the US, and prognosis is poor with a 5-year survival of 35.7%.^1^ The most common brain cancer is glioblastoma, which is responsible for 50.1% of cases.^1^ Given the low incidence of this form of cancer, there is limited evidence regarding potential risk factors. The risk of brain cancer has been noted to be higher in males, increase with age, and differ based on race and ethnicity.^1^ A variety of genetic syndromes, such as Li-Fraumeni, neurofibromatosis, and tuberous sclerosis, have also been associated with brain cancers.^2^

Very little is known about the association between exposures and subsequent brain cancer risk, with the exception of ionizing radiation, which has been established to increase risk.^3,4,5^ One proposed exposure that might increase the risk of brain cancers is traumatic brain injury (TBI). However, the current body of evidence with respect to TBI and subsequent brain cancer is conflicting. Some studies have suggested that brain cancer is associated with prior TBI,^6,7,8,9^ while other studies have not observed such an association.^10,11,12,13^

A history of TBI is common in post-9/11 era veterans, with 20% who served in Iraq and Afghanistan experiencing a TBI in the course of their service.^14^ Furthermore, TBI has been associated with poor long-term outcomes in the veteran population, including cardiovascular disease,^15^ dementia,^16^ epilepsy,^17^ and poor mental health outcomes.^18^ However, to our knowledge, the potential association between TBI and brain cancer has not been examined in a veteran cohort. Therefore, we sought to examine the potential link between an episode of TBI (stratified by severity) and the subsequent development of brain cancer in a large cohort of post-9/11 era veterans.

## Methods

The research protocol was reviewed and approved by the University of Utah Institutional Review Board and was conducted in accordance with all applicable federal regulations. The requirement for informed consent was waived by the institutional review board in accordance with Common Rule 45 CFR §46.116(f). This study included veterans from the Long-Term Impact of Military-Relevant Brain Injury Consortium–Chronic Effects of Neurotrauma Consortium (LIMBIC-CENC) Phenotype Study. The LIMBIC-CENC consolidates data from a variety of Department of Veterans Affairs (VA) and Department of Defense (DoD) sources, including the VA/DoD Identity Repository, the VA Corporate Data Warehouse, the DoD and VA Infrastructure for Clinical Intelligence (DaVINCI), the Theatre Data Management Store, the DoD Trauma Registry, the VA comprehensive TBI evaluation, and the National Death Index. The study was conducted from October 1, 2004, to September 20, 2019. Data analysis was performed between January 1 and June 26, 2023. The Strengthening the Reporting of Observational Studies in Epidemiology (STROBE) reporting guideline was used in reporting this study. To be included, veterans had to have received health care for at least 3 years in the DoD and, for those who entered the VA, 2 years of VA care. Veterans were excluded if they had no encounters within the study period, had an index date outside the study period, had a diagnosis of brain cancer before the index date or outside the study period, died before the index date (non-TBI cohort), or had missing data for a variable of interest. Given earlier work suggesting reverse causality (ie, brain cancer resulting in an accident and TBI with subsequent brain cancer diagnosis),^10^ individuals who developed brain cancer within 1 year of their TBI were excluded.

The primary variable of interest was TBI severity, categorized as mild, moderate or severe (moderate/severe), or penetrating. Traumatic brain injury was defined by using a hierarchical approach that prioritized data from the Theatre Data Management Store and DoD Trauma Registry (Glasgow Coma Scale score, Abbreviated Injury Severity Score, and *International Classification of Diseases, Ninth Revision, Clinical Modification (ICD-9-CM)* or *International Statistical Classification of Diseases, Tenth Revision, Clinical Modification (ICD-10-CM)* (together, *ICD-9/10*), followed by self-reported loss of consciousness (≤30 minutes, mild; >30 minutes, moderate/severe), alteration of consciousness or posttraumatic amnesia (≥24 hours, mild; >24 hours, moderate/severe^19^) identified in the VA comprehensive TBI evaluation, followed by *ICD-9/10* diagnosis codes using the 2012 Armed Forces Health Surveillance System algorithm.^20^ The list of *ICD-9/10* codes used to diagnose TBI and TBI severity is reported in eTable 1 in Supplement 1.

Other covariates of interest that have been associated with the development of brain cancer (ie, age, sex, and self-reported race and ethnicity) and service-related factors that could serve as surrogates for environmental exposures (ie, service branch, rank, and military component) were obtained from the VA/DoD Identity Repository. Service branch was categorized as Air Force, Army, Marines, Navy/Coast Guard, and Other (Public Health Service, Merchant seaman, and Commissioned Corps of the National Oceanic and Atmospheric Administration). Rank was categorized as officer, warrant officer, and enlisted. Military component was categorized as Active Duty, Reserve, and National Guard.

The primary outcome of interest was brain cancer, which was defined using 2 methods. First, we examined *ICD-9/10* codes from DaVINCI (Table 1). Individuals were considered to have the diagnosis if they had either 1 inpatient encounter with an *ICD-9/10* code in any diagnostic position or at least 3 outpatient encounters within 90 days in the first or second diagnostic position. Second, we examined causes of death based on *ICD-10* codes from the National Death Index (Table 1).

**Table 1.** List of *ICD-9/10* Codes Used to Diagnose Brain Cancer

*ICD* edition	Code	Code definition
10	C710	Malignant neoplasm of cerebrum, except lobes and ventricles
10	C711	Malignant neoplasm of frontal lobe
10	C712	Malignant neoplasm of temporal lobe
10	C713	Malignant neoplasm of parietal lobe
10	C714	Malignant neoplasm of occipital lobe
10	C715	Malignant neoplasm of cerebral ventricle
10	C716	Malignant neoplasm of cerebellum
10	C717	Malignant neoplasm of brain stem
10	C718	Malignant neoplasm of overlapping sites of brain
10	C719	Malignant neoplasm of brain, unspecified
9	191.0	Malignant neoplasm of cerebrum, except lobes and ventricles
9	191.1	Malignant neoplasm of frontal lobe
9	191.2	Malignant neoplasm of temporal lobe
9	191.3	Malignant neoplasm of parietal lobe
9	191.4	Malignant neoplasm of occipital lobe
9	191.5	Malignant neoplasm of ventricles
9	191.6	Malignant neoplasm of cerebellum not otherwise specified
9	191.7	Malignant neoplasm of brain stem
9	191.8	Malignant neoplasm of other parts of brain
9	191.9	Malignant neoplasm of brain, unspecified

Abbreviation: *ICD-9/10*, *International Classification of Diseases, Ninth Revision, Clinical Modification *and *International Statistical Classification of Diseases, Tenth Revision, Clinical Modification.*

### Statistical Analysis

Standard descriptive statistical tests were performed to compare the baseline characteristics of veterans who did vs did not have an episode of TBI. We then used Fine-Gray competing risks models to evaluate the potential risk imparted by TBI on subsequent brain cancer accounting for the risk of death not associated with brain cancer. In addition to univariate analysis, we ran nested multivariable models to adjust for demographic characteristics including age at index date, sex, race and ethnicity, service branch, rank, and component in a nested fashion. These results are reported as subdistribution hazard ratios (HRs) with 95% CIs. For veterans with TBI, the index date used for analysis was defined as the date of a TBI diagnosis. For veterans without TBI, index dates were simulated by drawing from the distribution of true index dates within each age bracket as previously described.^21^ The primary outcome was brain cancer based on diagnosis codes (obtained from DaVINCI) or death from brain cancer (National Death Index). The competing risk for these models was death not associated with brain cancer. All tests were 2-tailed, and statistical significance was determined at an α level of .05. Statistical analysis was conducted using SAS Enterprise Guide, version 8.2 (SAS Institute).

## Results

**Figure 1.**
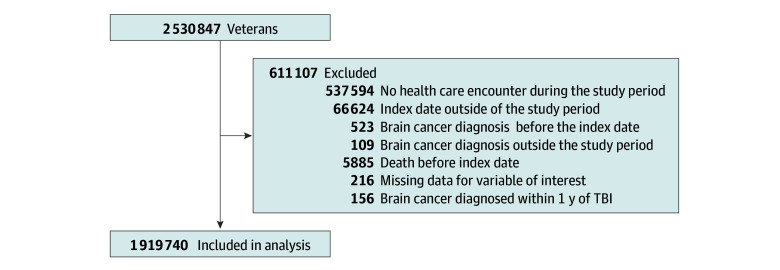
Development of the Study Cohort TBI indicates traumatic brain injury.

After the application of our inclusion and exclusion criteria, the total cohort included 1 919 740 veterans, most of whom were male (80.25% vs female [19.75%]) and non-Hispanic White (63.11%). Median age at the index date was 31 (IQR, 25-42) years. The cohort included 385 848 veterans with mild TBI, 46 859 with moderate/severe TBI, and 17 173 with penetrating TBI. The full LIMBIC-CENC cohort consists of 2 530 847 veterans. We excluded 21.4% of the potential population: 537 594 individuals who did not have a health care encounter during the study period, 66 624 of those who had an index date outside the study period, 523 with a brain cancer diagnosis before the index date, 109 with a brain cancer diagnosis outside the study period, 5885 who died before the index date, 216 with missing data for a variable of interest, and 156 with brain cancer diagnosed within 1 year of the index date (Figure 1).

Baseline characteristics comparing veterans with TBI (stratified by severity) with those without TBI are reported in Table 2. Significant differences between the groups were noted for all variables examined. Age at the index date was broadly similar, ranging from a median of 30 years in the mild TBI (IQR, 25-40 years) and moderate/severe TBI (IQR, 25-39 years) groups to 33 (IQR, (26-43) years in the penetrating TBI group. All TBI groups had a higher proportion of males (ranging from 86.67% in the mild TBI group to 88.42% in the penetrating TBI group) compared with the no TBI group (78.24%). White, non-Hispanic was the majority racial and ethnic group, accounting for 63.11% of the individuals, followed by the Black non-Hispanic group, accounting for 17.72% of the veterans. Black, non-Hispanic service members were less likely to have TBI exposure than White, non-Hispanic members. Army and Marine service members were more likely to have TBI exposure. The TBI groups also had higher proportions of enlisted personnel (86.96% [penetrating] to 91.41% [moderate/severe]) compared with the no TBI group (82.97%). As expected, brain cancer was a rare diagnosis, occurring in 0.02% of individuals in the no TBI and mild TBI groups. However, brain cancer was more common in individuals with moderate/severe TBI (0.04%). The absolute number of individuals with penetrating TBI and brain cancer was less than or equal to 10 (≤0.06%; below the minimum reportable cell size per our data-sharing agreements). Specific diagnostic codes for patients with brain cancer are reported in eTable 2 in Supplement 1. The crude incidence rates per 100 000 person-years were 3.06 (95% CI, 2.73-3.41) for no TBI, 2.85 (95% CI, 2.26-3.55) for mild TBI, 4.88 (95% CI, 2.84-7.81) for moderate/severe TBI, and 10.34 (95% CI, 4.73-19.63) for penetrating TBI (*P* < .001). Follow-up times were similar for no TBI (median, 7.2 years), mild and moderate/severe TBI (median, 7.4 years), but was less for penetrating TBI (median, 3.9 years), with an overall median of 7.2 (IQR, 4.1-10.1) years.

**Table 2.** Characteristics of the Study Cohort

Characteristic	No. (%)					*P* value
	Overall cohort (N = 1 919 740)	TBI				
		None (n = 1 469 860)	Mild (n = 385 848)	Moderate/severe (n = 46 859)	Penetrating (n = 17 173)	
Age, median (IQR), y	31 (25-42)	31 (24-43)	30 (25-40)	30 (25-39)	33 (26-43)	<.001
Sex						
Male	1 540 623 (80.25)	1 149 988 (78.24)	334 418 (86.67)	41 032 (87.56)	15 185 (88.42)	<.001
Female	379 117 (19.75)	319 872 (21.76)	51 430 (13.33)	5827 (12.44)	1988 (11.58)	
Race and ethnicity						
Asian and Pacific Islander	115 887 (6.04)	81 994 (5.58)	28 919 (7.49)	3711 (7.92)	1263 (7.35)	<.001
Black, Hispanic	10 366 (0.54)	8124 (0.55)	1954 (0.51)	211 (0.45)	77 (0.45)	
Black, non-Hispanic	340 182 (17.72)	266 045 (18.10)	64 633 (16.75)	6941 (14.81)	2563 (14.92)	
Hispanic	175 711 (9.15)	133 944 (9.11)	35 858 (9.29)	4247 (9.06)	1662 (9.68)	
American Indian	30 572 (1.59)	21 899 (1.49)	7463 (1.93)	899 (1.92)	311 (1.81)	
White, non-Hispanic	1 211 531 (63.11)	926 620 (63.04)	243 389 (63.08)	30 456 (64.99)	11 066 (64.44)	
Unknown	35 491 (1.85)	31 234 (2.12)	3632 (0.94)	394 (0.84)	231 (1.35)	
Service branch						
Army	898 076 (46.78) 404 347 (21.06)	625 299 (42.54)	234 924 (60.89)	27 980 (59.71)	9873 (57.49)	<.001
Air Force	360 269 (18.77)	353 372 (24.04)	43 522 (11.28)	5273 (11.25)	2180 (12.69)	
Navy/Coast Guard	254 555 (13.26)	304 254 (20.70)	47 946 (12.43)	5782 (12.34)	2287 (13.32)	
Marines	2493 (0.13)	184 715 (12.57)	59 236 (15.35)	7794 (16.63)	2810 (16.36)	
Other		2220 (0.15)	220 (0.06)	30 (0.06)	23 (0.13)	
Rank						
Enlisted	1 628 526 (84.83)	1 219 508 (82.97)	351 248 (91.03)	42 833 (91.41)	14 937 (86.98)	<.001
Officer	264 525 (13.78)	229 228 (15.60)	29 929 (7.76)	3497 (7.46)	1871 (10.90)	
Warrant Officer	26 689 (1.39)	21 124 (1.44)	4671 (1.21)	529 (1.13)	365 (2.13)	
Component						
Active duty	1 077 647 (56.14)	818 905 (55.71)	218 201 (56.55)	28 265 (60.32)	12 276 (71.48)	<.001
Reserve	627 576 (32.69)	495 519 (33.71)	116 138 (30.10)	12 684 (27.07)	3235 (18.84)	
National Guard	214 517 (11.17)	155 436 (10.57)	51 509 (13.35)	5910 (12.61)	1662 (9.68)	
Follow-up time from index date, median (IQR), y	7.2 (4.1-10.1)	7.2 (4.1-10.1)	7.4 (4.4-10.2)	7.4 (4.5-10.7)	3.9 (2.0-7.8)	<.001
Brain cancer diagnosis	≤425 (0.02)	318 (0.02)	80 (0.02)	17 (0.04)	≤10 (≤0.06)	.008

Abbreviation: TBI, traumatic brain injury.

**Figure 2.**
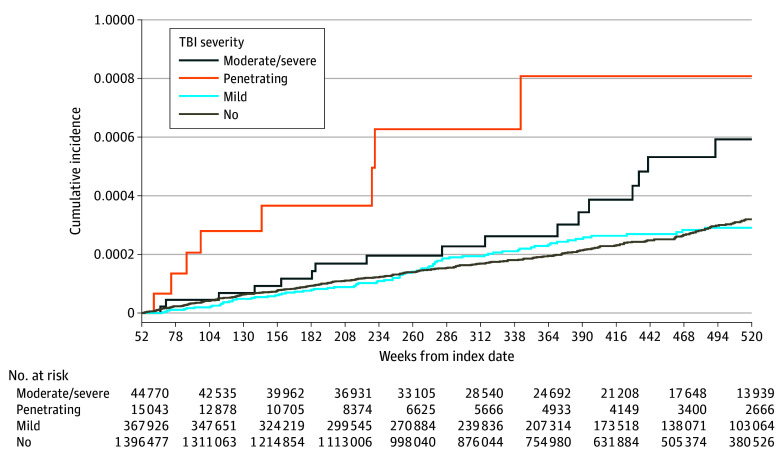
Cumulative Incidence Functions for the Outcome of Malignant Brain Cancer Stratified by Traumatic Brain Injury Severity TBI indicates traumatic brain injury.

The results from our nested models are reported in Table 3. On bivariate analysis, only penetrating TBI was associated with subsequent brain cancer (HR, 3.37; 95% CI, 1.74-6.53; *P* < .001). Moderate/severe TBI was not associated with the outcome in the bivariate analysis, but was significant in all of the other models examined, with adjusted HRs [AHRs] ranging from 1.79 to 1.90. Mild TBI was not associated with brain cancer in any of the models. In the fully adjusted model, penetrating TBI (AHR, 3.33; 95% CI, 1.71-6.49; *P* < .001) and moderate/severe TBI (AHR, 1.90; 95% CI, 1.16-3.12; *P* = .01), but not mild TBI (AHR, 1.14; 95% CI, 0.88-1.47; *P* = .31), were associated with subsequent brain cancer. This association is presented in Figure 2, showing a higher incidence among service members with moderate/severe TBI and penetrating TBI exposure compared with service members without TBI. We assessed the proportionality assumption using Schoenfeld residuals, which indicated that, for the TBI exposure variable, the risk of brain cancer increased over time. We chose to report HRs without time interactions as they represent a more conservative estimate of long-term risk of brain cancer in our data. Results of models including time by TBI exposure interaction term are included in eTable 3 in Supplement 1.

**Table 3.** Bivariate and Multivariable Competing Risk Models for the Outcome of Brain Cancer

Model	TBI severity^a^					
	Mild		Moderate/severe		Penetrating	
	HR (95% CI)	*P* value	HR (95% CI)	*P* value	HR (95% CI)	*P* value
Bivariate	0.92 (0.72-1.18)	.53	1.56 (0.96-2.55)	.07	3.37 (1.74-6.53)	<.001
Model 1^b^	1.06 (0.83-1.36)	.63	1.82 (1.11-2.97)	.02	3.34 (1.73-6.46)	<.001
Model 2^c^	1.03 (0.83-1.36)	.65	1.79 (1.10-2.93)	.02	3.28 (1.69-6.34)	<.001
Model 3^d^	1.14 (0.88-1.47)	.31	1.90 (1.16-3.12)	.01	3.33 (1.71-6.49)	<.001

Abbreviations: HR, hazard ratio; TBI, traumatic brain injury.

^a^ Compared with participants without TBI.

^b^ Adjusted for age.

^c^ Adjusted for age, sex, and race and ethnicity.

^d^ Adjusted for age, sex, race and ethnicity, service branch, rank, and component.

## Discussion

While brain cancer is an uncommon diagnosis, it is a devastating one, with very few known risk factors. We found that moderate/severe and penetrating TBI, but not mild TBI, were associated with the subsequent development of brain cancer. This finding suggests that, while relatively mild TBI may not increase the risk, more severe TBI may predispose injured veterans to subsequent brain cancer.

While some earlier work has reported an association between TBI and subsequent brain cancer,^6,7,8,9^ the 3 largest epidemiologic studies done to date have not found an association.^10,11,12^ The first of these, by Inskip and colleagues,^10^ examined 228 055 Danish residents who were hospitalized with a head injury and did not observe an association between TBI and glioma. They reported an incidence ratio of 1.15 (95% CI, 0.99-1.32), which likely would have been statistically significant with a larger sample size comparable with ours. In contrast to our work, the results were not stratified by TBI severity. It is likely, however, that most of the individuals had mild TBI, since 71% were classified as concussion. In our study, we did not see an association with mild TBI and subsequent brain cancer. It is therefore possible that the absence of an association seen in the Inskip et al^10^ study was due to the inclusion of these relatively low-risk patients. The second study, by Munch and colleagues,^11^ evaluated a large cohort of individuals in Denmark and examined the association between structural brain injury, of which TBI was 1 subtype, and the subsequent diagnosis of stage III/IV astrocytomas. These authors excluded mild TBI. In contrast to our work, however, they did not find an association between TBI and brain cancer after the exclusion of malignant neoplasms diagnosed within 1 year of the TBI. However, they noted a trend toward increased risk from 1 to 4 years after injury with a relative risk of 1.99. In comparison with this work, our study included a larger number of individuals with TBI (449 880 vs 48 194), with a longer follow-up period and a larger number of events. It is therefore possible that the results from the Munch et al^11^ study represent type II error. Other important differences with the present study include older age (median, 51 years in Munch et al^11^) and different administrative code definitions for TBI. In the third study, Nygren and colleagues^12^ examined a large Swedish cohort that included 311 006 individuals with TBI and 161 with an astrocytoma diagnosis. After a mean follow-up of 10.4 years, the authors did not note an increased risk of brain cancer with TBI. While the authors stratified by severity, their definitions were less granular than our definitions. These included concussion, severe head trauma with no neurosurgery, and severe head trauma with neurosurgery. Furthermore, while only the *ICD-9* codes can be directly compared with our definition, we included substantially more codes, which likely increased our sensitivity to identify TBI. Taken together, the earlier epidemiologic work is limited by less granular TBI definitions and low event rates, and risk may be diluted by mild TBI, which we observed not to be associated with brain cancer. Therefore, our results implying that only moderate/severe or penetrating TBI is associated with subsequent brain cancer are potentially consistent with this prior body of work and add to our understanding of risk after TBI. However, it is possible that our findings may also be TBI by military exposure interactions, where the risk is higher among TBI with a greater likelihood of blood-brain barrier breech (moderate/severe and penetrating injuries).

A link between TBI and subsequent brain cancer has been recognized as biologically plausible for some time. In 1978, Morantz and Shain^22^ examined a rat model for neural tumors and found that animals exposed to a cerebral stab wound were more likely to develop gliomas compared with uninjured control animals. Subsequently, a variety of potential mechanisms have been proposed, including alterations in metabolism, inflammation, astrocyte proliferation, and stem cell migration and differentiation.^23,24,25^ More recently, Richards and colleagues^26^ performed single-cell RNA sequencing on glioblastoma stem cells from 26 individuals. They found that these cells mapped to states reminiscent of neural development and the inflammatory wound response. Based on these results, the authors postulated that glioblastoma may arise as a response to injury, such as TBI, in patients with a mutated genomic background.

### Strengths and Limitations

Given the rarity of brain cancer diagnoses, the main strength of our study was the large size of the cohort, which was followed up for an extended period of time. However, there are several limitations to the present study. First, we were limited to *ICD-9/10* codes and did not have access to pathologic diagnoses. Second, many veterans were excluded from analysis (24.1%). While the predominate reason for exclusion was no health care encounter during the study period, 156 individuals were excluded due to brain cancer diagnosis within a year of the TBI diagnosis. Some TBI diagnoses are identified by screening and clinical history—sometimes years after the injury in the case of VA TBI screening. Thus, our results are likely a conservative estimate. Third, we were not able to control for other potential confounders due to a lack of available data, particularly on potential toxic exposures that might have increased the risk of brain cancer. Fourth, TBIs that were diagnosed and treated outside the DoD and VA health care systems were not captured. Fifth, the results from this predominantly young, male military population may not be generalizable to the US population as a whole. For example, there was an insufficient number of events in female patients to conduct a stratified analysis based on sex.

## Conclusions

In this cohort study of post-9/11 era veterans, we found that moderate/severe TBI and penetrating TBI were associated with the subsequent development of brain cancer. While the absolute number of brain cancer diagnoses was small, these diagnoses are associated with profoundly poor outcomes. Given that TBI is a common injury incurred in the course of military service, further research of this rare but devastating condition is needed to better identify those at risk and develop screening protocols.

## Notes

Supplement 1. **eTable 1.** *International Classification of Diseases 9th and 10th Edition Clinical Modification* Codes Used to Determine Traumatic Brain Injury **eTable 2.** Brain Cancer Codes Among Patients With a Brain Cancer Diagnosis **eTable 3.** Results of Competing Risk Regression Model With TBI by Log Time Interaction to Assess Nonproportionality

Supplement 2.
Data Sharing Statement
