# Supplementary material for: Traumatic Brain Injury and Subsequent Risk of Brain Cancer in US Veterans of the Iraq and Afghanistan Wars
Source: JAMA Netw Open. 2024 Feb 15;7(2):e2354588. doi: 10.1001/jamanetworkopen.2023.54588 (PMC10870183; doi:10.1001/jamanetworkopen.2023.54588)
Supplement: Supplement 1. — eTable 1. International Classification of Diseases 9th and 10th Edition Clinical Modification Codes Used to Determine Traumatic Brain Injury eTable 2. Brain Cancer Codes Among Patients With a Brain Cancer Diagnosis eTable 3. Results of Competing Risk Regression Model With TBI by Log Time Interaction to Assess Nonproportionality [file jamanetwopen-e2354588-s001.pdf]

Supplementary Online Content

Stewart IJ, Howard JT, Poltavskiy E, et al. Traumatic brain injury and subsequent risk of brain cancer in veterans of the Iraq and Afghanistan Wars. *JAMA Netw Open*. 2024;7(2):e2354588. doi:10.1001/jamanetworkopen.2023.54588

eTable 1. *International Classification of Diseases 9th and 10th Edition Clinical Modification* Codes Used to Determine Traumatic Brain Injury

eTable 2. Brain Cancer Codes Among Patients With a Brain Cancer Diagnosis

eTable 3. Results of Competing Risk Regression Model With TBI by Log Time Interaction to Assess Nonproportionality

This supplementary material has been provided by the authors to give readers additional information about their work.

© 2024 Stewart IJ et al. *JAMA Network Open.*

eTable 1. *International Classification of Diseases 9th and 10th Edition Clinical Modification* Codes Used to Determine Traumatic Brain Injury

ICD-9	ICD-10
<u>Mild</u><br>80000, 80001, 80002, 80006, 80009, 80050, 80051, 80052, 80100, 80101, 80102, 80106, 80109, 80150, 80151, 80152, 80300, 80301, 80302, 80306, 80309, 80350, 80351, 80352, 80400, 80401, 80402, 80406, 80409, 80450, 80451, 80452, 8500, 8501, 85011, 3102, 95901, 850, 8505, 8509<br><br><u>Moderate</u><br>80003, 80010, 80011, 80012, 80013, 80016, 80019, 80020, 80021, 80022, 80023, 80026, 80029, 80030, 80032, 80031, 80033, 80036, 80039, 80040, 80041, 80042, 80043, 80046, 80049, 80053, 80056, 80059, 80103, 80110, 80111, 80112, 80113, 80116, 80119, 80120, 80121, 80122, 80123, 80126, 80129, 80130, 80131, 80132, 80133, 80136, 80139, 80140, 80141, 80142, 80143, 80146, 80149, 80153, 80156, 80159, 80303, 80310, 80311, 80312, 80313, 80316, 80319, 80320, 80321, 80322, 80323, 80326, 80329, 80330, 80331, 80332, 80333, 80336, 80339, 80340, 80341, 80342, 80343, 80346, 80349, 80353, 80356, 80359, 80403, 80410, 80411, 80412, 80413, 80416, 80419, 80420, 80421, 80422, 80423, 80426, 80429, 80430, 80431, 80432, 80433, 80436, 80439, 80440, 80441, 80442, 80443, 80446, 80449, 80453, 80456, 80459, 85012, 8502, 85100, 85101, 85102, 85103, 85106, 85109, 85120, 85121, 85122, 85123, 85126, 85129, 85140, 85141, 85142, 85143, 85146, 85149, 85160, 85161, 85162, 85163, 85166, 85169, 85180, 85181, 85182, 85183, 85186, 85189, 85200, 85201, 85202, 85203, 85206, 85209, 85220, 85221, 85222, 85223, 85226, 85229, 85240, 85241, 85242, 85243, 85246, 85249, 85300, 85301, 85302, 85303, 85306, 85309, 85401, 85402, 85403, 85406, 85409	<u>Mild</u><br>F0781, S020XXA, S020XXB, S0210XA, S0210XB, S0291XA, S0291XB, S060X0A, S060X1A, S060X9A, S098XXA, S0990XA<br><br><u>Moderate</u><br>S020XXA, S020XXB, S0210XA, S0210XB, S0291XA, S0291XB, S060X0A, S060X2A, S060X3A, S060X4A, S061X0A, S061X1A, S061X2A, S061X3A, S061X4A, S061X9A, S06330A, S06331A, S06332A, S06333A, S06334A, S06339A, S06360A, S06361A, S06362A, S06363A, S06364A, S06369A, S06370A, S06371A, S06372A, S06373A, S06374A, S06379A, S06380A, S06381A, S06382A, S06383A, S06384A, S06389A, S064X0A, S064X1A, S064X2A, S064X3A, S064X4A, S064X9A, S065X0A, S065X1A, S065X2A, S065X3A, S065X4A, S065X9A, S066X0A, S066X1A, S066X2A, S066X3A, S066X4A, S066X9A, S06890A, S06891A, S06892A, S06893A, S06894A, S06899A, S069X0A, S069X1A, S069X2A, S069X3A, S069X4A, S069X9A<br><br><u>Severe</u><br>S020XXA, S020XXB, S0210XA, S0210XB, S0291XA, S0291XB, S060X5A, S060X6A, S061X5A, S061X6A, S061X7A, S061X8A, S06335A, S06336A, S06337A, S06338A, S06365A, S06366A, S06367A, S06368A, S06375A, S06376A, S06377A, S06378A, S06385A, S06386A, S06387A, S06388A, S064X5A, S064X6A, S064X7A, S064X8A, S065X5A, S065X6A, S065X7A, S065X8A, S066X5A, S066X6A, S066X7A, S066X8A, S06895A, S06896A, S06897A, S06898A, S069X5A, S069X6A, S069X7A, S069X8A

© 2024 Stewart IJ et al. *JAMA Network Open.*

ICD-9	ICD-10
Severe<br>80004, 80005, 80014, 80015, 80024, 80025, 80034, 80035, 80044, 80045, 80054, 80055, 80104, 80105, 80114, 80115, 80124, 80125, 80134, 80135, 80144, 80145, 80154, 80155, 80304, 80305, 80314, 80315, 80324, 80325, 80334, 80335, 80344, 80345, 80354, 80355, 80404, 80405, 80414, 80415, 80424, 80425, 80434, 80435, 80444, 80445, 80454, 80455, 8503, 8504, 85104, 85105, 85124, 85125, 85144, 85145, 85164, 85165, 85184, 85185, 85204, 85205, 85224, 85225, 85244, 85245, 85304, 85305<br>85404, 85405<br><br>Penetrating<br>8006X, 8007X, 8008X, 8009X, 8016X, 8017X, 8018X, 8019X, 8036X, 8037X, 8038X, 8039X<br>8046X, 8047X, 8048X, 8049X, 8511X, 8513X, 8515X, 8517X, 8519X, 8521X, 8523X, 8525X,<br>8531X, 8541X))<br><br>Unclassified<br>9501, 9502, 9503, 9070, 85400<br><br>Personal History of Traumatic Brain Injury<br>V15.52	Penetrating<br>S0190XA, S020XXB, S0210XB, S0291XB, S06330A, S06331A, S06332A, S06333A, S06334A, S06335A, S06336A, S06337A, S06338A, S06339A, S06360A, S06361A, S06362A, S06363A, S06364A, S06365A, S06366A, S06367A, S06368A, S06369A, S06370A, S06371A, S06372A, S06373A, S06374A, S06375A, S06376A, S06377A, S06378A, S06379A, S06380A, S06381A, S06382A, S06383A, S06384A, S06385A, S06386A, S06387A, S06388A, S06389A, S064X0A, S064X1A, S064X2A, S064X3A, S064X4A, S064X5A, S064X6A, S064X7A, S064X8A, S064X9A, S065X0A, S065X1A, S065X2A, S065X3A, S065X4A, S065X5A, S065X6A, S065X7A, S065X8A, S065X9A, S066X0A, S066X1A, S066X2A, S066X3A, S066X4A, S066X5A, S066X6A, S066X7A, S066X8A, S066X9A, S06890A, S06891A, S06892A, S06893A, S06894A, S06895A, S06896A, S06897A, S06898A, S06899A, S069X0A, S069X1A, S069X2A, S069X4A, S069X5A, S069X6A, S069X7A, S069X8A, S069X9A<br><br>Unclassified<br>S0402XA, S04039A, S04049A, S06890A, S069X9S<br><br>Personal History of Traumatic Brain Injury<br>Z87820

© 2024 Stewart IJ et al. *JAMA Network Open.*

eTable 2. Brain cancer codes among patients with a brain cancer diagnosis

ICD 9	ICD 10	Code Definition	N (%)
191.9	C719	Malignant neoplasm of brain, unspecified	219 (51.7)
191.1	C711	Malignant neoplasm of frontal lobe	63 (14.9)
191.2	C712	Malignant neoplasm of temporal lobe	38 (9.0)
191.3	C713	Malignant neoplasm of parietal lobe	31 (7.3)
191.6	C716	Malignant neoplasm of cerebellum	22 (5.2)
191.0	C710	Malignant neoplasm of cerebrum, except lobes and ventricles	18 (4.2)
191.8	C718	Malignant neoplasm of overlapping or other sites of brain	14 (3.3)
191.7	C717	Malignant neoplasm of brain stem	11 (2.6)
191.4	C714	Malignant neoplasm of occipital lobe	≤10 (≤2.4)
191.5	C715	Malignant neoplasm of cerebral ventricle	≤10 (≤2.4)

ICD: International Classification of Diseases

© 2024 Stewart IJ et al. *JAMA Network Open.*

eTable 3. Results of competing risk regression model with TBI by log time interaction to assess nonproportionality

**Factor**	**HR (95% CI); p value**
**TBI Status**	
Mild TBI	0.03 (0.01-0.05); <0.001
Moderate/Severe TBI	0.01 (0.00-0.01); <0.001
Penetrating TBI	0.00 (0.00-0.01); <0.001
**Logged follow-up time**	0.34 (0.32-0.36); <0.001
**Logged follow-up time * TBI Status Interaction**	
Mild TBI	1.59 (1.48-1.7); <0.001
Moderate/Severe TBI	2.05 (1.87-2.25); <0.001
Penetrating TBI	2.44 (2.20-2.72); <0.001

*Model adjusted for age, sex, race and ethnicity, service branch, rank, component, and marital status

© 2024 Stewart IJ et al. *JAMA Network Open.*
