# Supplementary material for: Traumatic Brain Injury and Subsequent Risk of Brain Cancer in US Veterans of the Iraq and Afghanistan Wars
Source: JAMA Netw Open. 2024 Feb 15;7(2):e2354588. doi: 10.1001/jamanetworkopen.2023.54588 (PMC10870183; doi:10.1001/jamanetworkopen.2023.54588)
Supplement: Supplement 2. — Data Sharing Statement [file jamanetwopen-e2354588-s002.pdf]

Data Sharing Statement

Stewart. Traumatic Brain Injury and Subsequent Risk of Brain Cancer in US Veterans of the Iraq and Afghanistan Wars. *JAMA Netw Open*. Published February 15, 2024. doi:10.1001/jamanetworkopen.2023.54588

Data
Data available: No

Additional Information
Explanation for why data not available: The authors do not control access to the data, therefore we cannot offer to share data. The data are owned by the VA and DoD and require permisions to access. We would however be willing to advise others on how to go through the process of aquiring these permisions.
